# Supplementary material for: Impact of high-speed rail on tourism in China
Source: PLoS One. 2022 Dec 8;17(12):e0276403. doi: 10.1371/journal.pone.0276403 (PMC9731443; doi:10.1371/journal.pone.0276403)
Supplement: S1 Table — (DOCX) [file pone.0276403.s001.docx]

**S1 Table. Abbreviation comparison table.**

| **No.** | **Abbreviations** | **Abbreviations Full name** | **Proxy indicators** |
| --- | --- | --- | --- |
| 1 | *TR* | Tourism revenue | Total tourism revenue of the city |
| 2 | *TA* | Tourist arrivals | Total tourist arrivals of the city |
| 3 | *DTR* | Domestic tourism revenue | Domestic tourism revenue of the city |
| 4 | *DTA* | Domestic tourist arrivals | Domestic tourist arrivals of the city |
| 5 | *HSR* | HSR opening | Dummy variable indicating whether the city has a HSR link |
| 6 | *Node* | Node level | Dummy variable indicating whether the city has more than one HSR line |
| 7 | *FASC* | Food and accommodation supply capacity | The number of star-rated hotels in the city |
| 8 | *SSC* | Sightseeing supply capacity | The number of China’s 5A scenic spots in the city |
| 9 | *ES* | Economic scale | Gross regional product of the city |
| 10 | *FE* | Fiscal expenditure | Local government expenditure of the city |
| 11 | *TCD* | Transportation convenience degree | Highways passenger traffic of the city |
| 12 | *IS* | Industrial structure | The weighted sum of the added value of the three industries of the city |
| 13 | *SIS* | Service industry support | The number of employed persons in the tertiary industry of the city |
| 14 | *PS* | Population size | The resident population of the city |
| 15 | *IL* | Income level | The average wage of employed persons in the city |
| 16 | *OD* | Opening degree | The proportion of foreign direct investment in the gross domestic product of the city |
