# Supplementary material for: Impact of high-speed rail on tourism in China
Source: PLoS One. 2022 Dec 8;17(12):e0276403. doi: 10.1371/journal.pone.0276403 (PMC9731443; doi:10.1371/journal.pone.0276403)
Supplement: Attachment — Submitted filename: Response to Reviewers.docx [file pone.0276403.s003.docx]

Dear editor:

Thank you for your kind letters of “PLOS ONE Decision: Revision required [PONE-D-22-18625R1]” on 06-Sep-2022, and for the reviewers’ comments concerning our manuscript entitled “Impact of high-speed rail on tourism in China”. These comments are of great help in improving the quality of our papers. We revised the manuscript in accordance with the editor’s and reviewers’ comments, and carefully proof-read the manuscript to minimize typographical, grammatical, and bibliographical errors.

Here below is our description on revision according to the reviewers’ comments. (The review comments are in black, and our response notes are in purple)

Reviewer 2

(1) Line 103-114. The research should highlight the differences with the existing research in terms of research object and research scope, so as to further clarify the innovation of selecting 286 cities in China.

The authors’ Answer: Thank you for your kind comment. As you said, the selection of 286 cities in China is also one of the innovative contributions of this paper. By reviewing the existing literature, we found that most of the literature takes a single HSR line as the research object to analyze its impact on the development of urban tourism, which may lead to the following two problems: first, different HSR lines are built in different regions, and the research conclusions may imply obvious regional characteristics. Second, the additive effect of multiple HSR lines may be wrongly attributed to the effect of the one HSR that is the subject of the study (Line108-111). In this paper, we collated data on all HSR line in China since 2008 and conducted empirical tests on a sample of 286 cities in China to avoid possible regional limitations of the study findings and estimation bias caused by the omission of cross lines (Line 115-118). As suggested, we have added the supplementary explanation for this innovation in the Introduction section.

(2) Comparison with previous studies should be given, including the advance of the methods and the accuracy of the conclusions. It is essential to claim the novelty of the article.

The authors’ Answer: Thank you for your kind comment. In the discussion section, we have compared our results with existing literature and claimed our innovative contribution (Line 534-548). As suggested, we improved this part and refined some content to add to the Introduction section (Line 118-121). **First**, most of the existing literature focuses on the horizontal effect of high-speed rail on economic growth, ignoring the rate effect, which may lead to an overestimation of the effect of high-speed rail. Our study makes up for this by confirming the existence of a rate effect. However, we do not give a clear answer to the question that the role of high-speed rail may be overestimated. After comparing our estimation results with Zeng and Chen’s [1], we found that the rate effect is indeed lower than the level effect, and we added these in the revised draft. **Second**, the heterogeneity of the existing literature on high-speed rail is attributed to differences in the socioeconomic characteristics of cities, ignoring the impact of differences in high-speed rail itself. This is not conducive to an accurate understanding of the role of high-speed rail in network operation. Our study breaks this limitation and confirms the existence of the node effect. **Third**, we analyze the comparative advantages of each city in tourism specialization based on node effect, which provides a new perspective to explain the heterogeneous impact of high-speed rail on tourism development.

(3) Can spatial compression be expressed by Figures?

The authors’ Answer: Thank you for your kind comment. As suggested, we draw a schematic figure of the time-space compression effect of HSR (Fig 1) (Line 140-141), add relevant explanations (Line 131-135) and adjusted the serial number of figures in the article. When the spatial distance between cities is fixed, the opening of HSR shortens the commuting time between cities on the basis of improving the accessibility of cities (Fig 1a). When the commuting time to the destination city is fixed, the opening of HSR makes the spatial structure between cities more compact (Fig 1b).

2h

1h

(a) Time compression

A

B

A

B

A

(b) Space compression

2h traffic circle

1h traffic circle

A

After HSR opening

After HSR opening

B

B

2h traffic circle

1h traffic circle

**Fig 1. The time-space compression effect of HSR.**

(4) Move the abbreviations either at the beginning or end of the article.

The authors’ Answer: Thank you for your kind comment. In the last revision of the manuscript, we added a column on the meaning of abbreviations in Table 1 to make the meaning of each abbreviation clearer to the reader (Line 287). But that may still not be intuitive enough. We have carefully considered your comments. In accordance with the principle of "Figures and Tables follow the text", we did not move Table 1, but we also made the following two improvements. **First**, from Table 2 to Table 6, we explain the abbreviations in the table one by one in the form of table notes (Line 313-315, Line 404-407, Line 440-443, Line 452-455, Line 497-501), such as “ES = Economic scale; FE = Fiscal expenditure; TCD = Transportation convenience degree; IS = Indus-trial structure; SIS = Service industry support; PS = Population size; IL = Income level; OD = Opening degree.”. Second, we made a comparison table of abbreviations (S1 Table) and put it at the end of the article as Supporting information (Line 724-725). The table lists all abbreviations, their corresponding full names and the proxy indicators used.

**S1 Table. Abbreviation comparison table.**

| **No.** | **Abbreviations** | **Abbreviations Full name** | **Proxy indicators** |
| --- | --- | --- | --- |
| 1 | *TR* | Tourism revenue | Total tourism revenue of the city |
| 2 | *TA* | Tourist arrivals | Total tourist arrivals of the city |
| 3 | *DTR* | Domestic tourism revenue | Domestic tourism revenue of the city |
| 4 | *DTA* | Domestic tourist arrivals | Domestic tourist arrivals of the city |
| 5 | *HSR* | HSR opening | Dummy variable indicating whether the city has a HSR link |
| 6 | *Node* | Node level | Dummy variable indicating whether the city has more than one HSR line |
| 7 | *FASC* | Food and accommodation supply capacity | The number of star-rated hotels in the city |
| 8 | *SSC* | Sightseeing supply capacity | The number of China’s 5A scenic spots in the city |
| 9 | *ES* | Economic scale | Gross regional product of the city |
| 10 | *FE* | Fiscal expenditure | Local government expenditure of the city |
| 11 | *TCD* | Transportation convenience degree | Highways passenger traffic of the city |
| 12 | *IS* | Industrial structure | The weighted sum of the added value of the three industries of the city |
| 13 | *SIS* | Service industry support | The number of employed persons in the tertiary industry of the city |
| 14 | *PS* | Population size | The resident population of the city |
| 15 | *IL* | Income level | The average wage of employed persons in the city |
| 16 | *OD* | Opening degree | The proportion of foreign direct investment in the gross domestic product of the city |

References

1.	Zeng, Y.; Chen, J. The heterogeneous effect of high-speed rails on urban tourism development: An analysis based on the difference-in-differences approach. *Tourism Science* **2018**, *32*, 79-92, doi:10.16323/j.cnki.lykx.2018.06.006.

Sincerely yours,

Kehan Shi

Jinfang Wang

Xiaojin Liu

Xiaoying Zhao
