# Supplementary material for: Impact of high-speed rail on tourism in China
Source: PLoS One. 2022 Dec 8;17(12):e0276403. doi: 10.1371/journal.pone.0276403 (PMC9731443; doi:10.1371/journal.pone.0276403)
Supplement: Attachment — Submitted filename: Response to Reviewers.docx [file pone.0276403.s004.docx]

Dear editor:

Thank you for your kind letters of “PLOS ONE Decision: Revision required [PONE-D-22-18625R2]” on 19-Sep-2022, and for the reviewers’ comments concerning our manuscript entitled “Impact of high-speed rail on tourism in China”. These comments have been very helpful in improving the quality of our papers. Changes have been made in response to comments made by the reviewers.

Here below is our description on revision according to the reviewers’ comments. (The review comments are in black, and our response notes are in purple)

Reviewer 2

(1) The document presented is editorially hideous, such as Paragraph spacing(Introduction, Conclusions and discussion), Table expression, etc. It is the authors' responsibility to present their research in a clear and understandable way so that it can be used by all potential interested parties.

The authors’ Answer: We apologize for any problems with the typesetting of this document. In order to ensure that our manuscript meets PLOS ONE’s style requirements, we modified it according to the PLOS ONE style templates (http://journals.plos.org/plosone/s/file?id=ba62/PLOSOne_formatting_sample_title_authors_affiliations.pdf and http://journals.plos.org/plosone/s/file?id=wjVg/PLOSOne_formatting_sample_main_body.pdf).

First, we use the double-spaced paragraph format. Second, we set the text-indent to 1 character, except for the first paragraph below the head of each level. Third, we have modified the form of tables in our manuscript according to the PLOS ONE Table Guidelines (https://journals.plos.org/plosone/s/tables) by adding border-right and border-left.

In addition, we resize the affiliation font to 10pt and remove postal codes from the affiliation.

Sincerely yours,

Kehan Shi

Jinfang Wang

Xiaojin Liu

Xiaoying Zhao
